# How Chinese clinicians face ethical and social challenges in fecal microbiota transplantation: a questionnaire study

**DOI:** 10.1186/s12910-017-0200-2

**Published:** 2017-05-31

**Authors:** Yonghui Ma, Jinqiu Yang, Bota Cui, Hongzhi Xu, Chuanxing Xiao, Faming Zhang

**Affiliations:** 1 Center for Bioethics, Medical College, Xiamen University, Xiamen, China; 2 Department of Nursing, Medical College, Xiamen University, Xiamen, China; 3 Medical Center for Digestive Diseases, Second Affiliated Hospital of Nanjing Medical University, Nanjing, China; 4 Department of Gastroenterology, Xiamen Zhongshan Hospital Affiliated to Xiamen University, Xiamen, China

**Keywords:** Fecal microbiota transplantation, Research ethics, Informed consent, Risk benefit evaluation

## Abstract

**Background:**

Fecal microbiota transplantation (FMT) is reportedly the most effective therapy for relapsing *Clostridium Difficile* infection (CDI) and a potential therapeutic option for many diseases. It also poses important ethical concerns. This study is an attempt to assess clinicians’ perception and attitudes towards ethical and social challenges raised by fecal microbiota transplantation.

**Methods:**

A questionnaire was developed which consisted of 20 items: four items covered general aspects, nine were about ethical aspects such as informed consent and privacy issues, four concerned social and regulatory issues, and three were about an FMT bank. This was distributed to participants at the Second China gastroenterology and FMT conference in May 2015. Basic descriptive statistical analyses and simple comparative statistical tests were performed.

**Results:**

Nearly three quarters of the 100 respondents were gastro-enterologist physicians. 89% of all respondents believed FMT is a promising treatment modality for some diseases and 88% of whom chose clinical efficacy as the primary reason for recommending FMT. High expectation from patients and pressure on clinicians (33%) was reported as the most frequent reasons for not recommending FMT. The clinicians who had less familiarity with FMT reported significantly more worry related to the dignity and psychological impact of FMT compared to those who have high familiarity with FMT (51.6% vs 27.8%, *p* = 0.021).More than half of the respondents (56.1%) were concerned about the commercialization of FMT, although almost one in five respondents did not see this as a problem.

**Conclusions:**

We found most respondents have positive attitudes towards FMT but low awareness of published evidence. Informed consent for vulnerable patients, privacy and protection of donors were perceived as the most challenging ethical aspects of FMT. This study identified areas of limited knowledge and ways of addressing ethical issues and indicates the need to devise the education and training for clinicians on FMT.

**Electronic supplementary material:**

The online version of this article (doi:10.1186/s12910-017-0200-2) contains supplementary material, which is available to authorized users.

## Background

Health professionals and researchers frequently face ethical difficulties and uncertainties [1]. How they perceive, experience, and deal with them, and whether to seek ethics consultation services, have important implications on the quality and outcome of the healthcare service as well as the patients’ satisfaction. Sometimes this is reflected as increased complaints and litigation against healthcare practitioners. It is especially true for highly innovative interventions, which generally involve more uncertainties e.g. lack of adequate data on efficacy and safety, and risks in the long-term. In gastroenterology, Fecal Microbiota Transplantation (FMT) is such a case, though still in the investigational phase, it represents a promising and reportedly effective therapeutic alternative in treating CDI and many other diseases [2, 3]. The successful management of its ethical and social problems will affect the implementation of FMT in a responsible, sustainable, and ethically warranted way. This paper aims to examine clinician’s attitudes toward important ethical, social and regulatory issues surrounding FMT.

Fecal Microbiota Transplantation is the delivery of large amounts of intestinal microbiota (fecal suspension or purified fecal microbiota) from a pre-screened healthy donor into the intestinal tract of a patient, who usually has been cleared for treatment of a specific disease [4–6]. Its use was first documented for treating food poisoning and severe vomiting, diarrhea in ancient Traditional Chinese Medicine in the fourth century [7]. It is reportedly the most effective therapy for relapsing *Clostridium Difficile* infection (CDI) [5, 6, 8] and is also a potential treatment for gastrointestinal disorders and other diseases, including inflammatory bowel diseases (IBD), irritable bowel syndrome (IBS), obesity, diabetes, anorexia nervosa, food allergies, as well as neurodegenerative and neuro-developmental disorders [9–12]. Clinical trials are now underway to investigate the role of gastrointestinal microbiota in treating several of these conditions [13]. Although adequate evaluation of the efficacy and safety of this intervention is still lacking, there is increasing enthusiasm from doctors, regulatory agencies, policy makers, and patients for expanding its applications [14]. In addition, FMT is widely perceived as “natural ”or “organic” by patients, and therefore “safer” than conventional therapies such as antibiotics [15, 16], despite a few reports of potential complications [17]. As a ‘new’ treatment approach it runs the risk of being perceived as a panacea for a multitude of illnesses by the public, and extreme care must be taken to be precise in the use of terminology linked to this treatment approach and the indications for its use.

The U.S. Food and Drug Administration (USFDA) has chosen to regulate human feces as a biological product and drug [18]. This requirement aims to ensure safety, therapy standardization, and security of FMT. Regulating FMT in this way means that doctors are required to submit a time-consuming Investigational New Drug (IND) application before performing FMT. The FDA has subsequently waived the IND requirement only for treatment of recurrent CDI provided that the treating physician obtains adequate informed consent from the patient or their representative.

However, critics still have significant concerns about the social and ethical challenges raised by FMT [14, 19, 20], with numerous unanswered clinical and microbiological questions surrounding FMT. According to Ma et al. in discussion about ethics of fecal microbiota transplantation, immediate concerns include the selection and screening of donors, the vulnerability of patients, long-term safety and efficacy evaluation, informed consent. Other relevant considerations include the potential of FMT for commercial use and abuse, such as in health and longevity promotion based on the studies demonstrating the microbiota linked to aging [11, 21]. Moreover, in this direct-to-consumer era, the practice of patients posting their experiences and home-based “FMT DIY (do-it-yourself)“methods online is becoming increasingly common [13, 22].

Studies also suggested that there is a discrepancy between clinicians’ beliefs about FMT and patients willingness to accept FMT. On the one hand, despite its unappealing nature, patients and their families were open to considering it as a treatment alterative, especially when recommended by a physician [23], or even overwhelmingly willing to consider treatment with FMT [15] and eager for it to become available [16]. A recent study reported patients who have been actively seeking information and opportunity to receive FMT over a long time period and believing they could have benefited from undergoing FMT sooner [24]. On the other hand, physicians’ and gastroenterologists’ attitudes towards FMT are generally conservative or even negative [25], having poor health literacy on FMT [26], “limited experience” with FMT [27, 28], or will only consider when “scientifically justified and ethically approved” [29], which may reduce the likelihood that some patients receive information about FMT. In addition, whilst these discussions have been conducted amongst researchers and patients in North American and Europe, there has been paucity in the study of attitudes of clinicians in non-Western countries.

Those ethical challenges, as well as the discordance between clinicians’ and patients’ beliefs about FMT demonstrate a need for investigating clinicians’ perception and sensitivity to the awareness of and ethical issues related to FMT. Investigation of FMT is in constant and rapid evolution and development, consequently the treatment decisions based upon best evidence are sometimes difficult and might reflect healthcare quality problems and influence the patients’ satisfaction. These challenges must be addressed as part of a successful regulatory policy response to FMT and its effective implementation in practice.

This is the first study to examine clinician’s attitudes toward important ethical, social and regulatory issues surrounding FMT. Drawing on our early experiences performing FMT, establishing an FMT bank, and our familiarity with the literature in the field of FMT globally, we identified five major issues associated with FMT:

Patient consent and vulnerability,

Risk and safety,

Privacy and dignity,

Commercialization and.

Regulation.

In order to better understand the clinician’s awareness and perception of these issues, as well as the relevance of these issues to their practice, we surveyed attendees of the Second China Gastroenterology and FMT conference in May 9th, 2015.

## Methods

### Survey development

A short anonymous questionnaire survey (Additional file 1) was designed to obtain an overview of the general knowledge and attitudes towards the use of FMT of clinicians and associated team members, and specifically focused to assess their awareness and sensitivity to the identified ethical issues raised by FMT. The questionnaire consisted of four sections comprising 20 items: general knowledge and attitudes towards FMT (four items); perception of ethical concerns (nine items); belief about social and regulatory issues (four items); and views about FMT bank ethics (three items). Question formats included single choice, multiple-choice, and written short answers. A focus group study was conducted at the Institute of Digestive Endoscopy and Medical Center for Digestive Diseases at the Second Affiliated Hospital of Nanjing Medical University (Nanjing, China), including physicians and microbiologists who reviewed the questionnaire before distribution.

The questionnaires were distributed during the end of the sessions in the Second China Enterology and FMT conference in May, 2015 (Nanjing, China). This is a national conference all participants practiced medicine in China and all had some familiarity of FMT. The attendees were encouraged to fill in and return the questionnaires at the end of the conference.

### Study participants

The study participants were delegates at the above conference, and comprised of a variety of physicians, including gastroenterologists, infectious diseases, internal medicine, as well as Traditional Chinese Medicine practitioners, microbiologists, pharmacists, and nurses. Participants were informed in the description of the survey that their agreement to participate in the study was voluntary and completion constituted their informed consent. In this paper, we use “clinicians” and “participants” interchangeably to refer all the respondents, and do not making any differences.

### Ethical approval

The study protocol was reviewed and approved by two ethics committees: the Institutional Review Board of Medical College of Xiamen University and the Ethics Committees for Protection of Human Subjects of Second Affiliated Hospital of Nanjing Medical University.

### Analysis

Descriptive statistical analysis was used to analyze the participants’ answers to each item of the survey. The data obtained is presented as frequency counts and percentages by category. The general knowledge and attitude towards the use of FMT of the two groups (Group 1: participants who had performed FMT previously; Group 2: participants who had not performed FMT previously) were compared with the use of Pearson Chi-square test or Fisher’s exact test. All reported *P* values are two-sided, *P* < 0.05 was considered statistically significant. Data analysis was performed using the SPSS software system (SPSS for Windows, Version 17.0, SPSS Inc., Chicago, IL).

## Results

### Participants’ knowledge and attitudes towards FMT

In total 150 surveys were distributed and 109 were completed, nine of which were excluded because more than three questions were left unanswered. Of the 100 respondents who completed the questionnaires, 74 were gastroenterologists and internists, 17 were nurses, and five were microbiologists, one was a pharmacologist, and three were ‘other profession’. Overall, 89% had heard about FMT before attending the conference, 36% reported high familiarity with and had performed FMT previously. 89% respondents believe FMT is a promising treatment modality for some diseases, 6% were skeptical about the efficacy of FMT and 5% believe the current data is not sufficient to support the use of FMT. Of those having positive attitudes (*n* = 89) towards FMT, when asked if it is medically indicated and ethically approved, 82 (92.1%) were willing to recommend their patients for FMT, compared to 14 (14.3%) undecided who believed “it depends” on specific circumstances (such as failure in standard treatment), and 2 (2%) who would not recommend FMT whatsoever.

### Participants’ perception of ethical concerns

#### Participants’ opinions on informing patients of FMT

Of 82 respondents who indicated recommending FMT and 14 who believed potentially recommending FMT for patients (*n* = 96), clinical efficacy was the most cited reason (88%) for recommending FMT, patients being informed about aspects of efficacy. Other reasons for clinicians recommending FMT and notifying patients are: safety (64%), “natural” and “organic” (30%), failure of conventional treatment (20%), and 11% avoidance of antibiotics. In contrast, of 16 clinicians in either disagree (*n* = 2) or “it depends” groups(*n* = 14), the three most frequent reasons for not recommending FMT were: high expectation from patients and pressure from patients and media reports on clinicians to perform FMT(33%), long-term risk and safety unknown (27%), unproven treatment and unknown mechanism (22%), infections (19%), and non-standard treatment, increasing likelihood of medical litigation(14%) (Table 1).

**Table 1:** Clinicians’opinions on informing patients about FMT

(1) Possible reasons for recommending FMT which will inform patients are (*n* = 96):	
Clinical efficacy	88%
Safety	64%
Failure of conventional treatment	20%
“natural” and “organic”	30%
Avoidance of antibiotics	11%
(2) Possible reasons for not recommending FMT and will inform patients are (*n* = 16):	
Unproven treatment and unknown mechanism	22%
Long-term risk and safety unknown	27%
Infections	19%
High expectation from patients and pressure on physicians	33%
Not standard treatment, easily cause medical litigation	14%
(3) Which statement do you agree with regarding media portrayal of FMT as “magic” and “miracle” (*n* = 100)?	
This will mislead patients to unrealistic high expectations and neglect risk	70%
This would not affect patients as they are capable of making autonomous decisions	14%
I do not care, anyway patients would be informed by the informed consent form	16%

When asked to consider if the media have exaggerated the effect and downplayed the risk by describing FMT as “magic” and “miracle” therapy, and the likely impact on patient decision making, 70% of all respondents predicted that this will mislead patients to expect unrealistically high successful outcomes and neglect the risk. 14% predicted that this would not affect patients, as they are capable of making autonomous decisions, 16% reported patients would be informed by the informed consent form (Table 1).

#### Participants’ perceptions of risk, privacy, stigma issues

After reading a summary of information regarding risks of FMT, including some physical adverse events, e.g. fever, abdominal cramping or constipation, elevation of C-reative protein (CRP) [11], unknown infections, as well as the potential for FMT-induced mental illness (transmission of anxiety and depression) [30, 31], imbalance of neurotransmitters [32] (informed by advancements in the field of microbiome-gut-brain axis in recent years), the majority (71% of all 100 respondents) reported they would inform patients of all known and possible risks (physical, mental, cognitive, behavioral) and let them make the decision, 20% reported the notification level of risk depends on the comprehensive capacity of patients, 9% will only inform patients about the physical risk.

When asked if undergoing FMT will have a negative impact on patient dignity and psychological wellbeing (e.g. feelings of embarrassment, degradation, and stigma), out of all respondents, 43% agreed while 47% disagreed, and 10% ‘do not know’. The clinicians who had less familiarity with FMT reported significantly more worry about dignity and psychological issues compared to those who have high familiarity with FMT (51.6% vs 27.8%, *p* = 0.021).When asked about the administration method (if both are clinically feasible) and its impact on dignity, 68% preferred colonoscopic enteral tubing(TET) [33] while nasoduodenal tube delivery was preferred by only 32%.

The perceived measures for patients’ privacy protection are shown in Table 2. The majority of respondents (61%) believed establishing a standardized fecal microbiota bank is most effective, 42% believed safeguarding confidentiality of patient information during communication with colleagues and other patients, followed by anonymity (15%) and private rooms for treatment (15%).

**Table 2:** Participants’ perceived measures to respect and protect privacy

Participants’ perceived measures to respect and protect privacy are (*n* = 100):	
The donor and recipient should be kept anonymous to each other	22%
The donor should be kept anonymous	15%
Establishing standardized FMT bank	61%
Patients who will undergo FMT should have private rooms for treatment	15%
Safeguarding confidentiality of patient information during communication with colleagues and other patients	42%

#### Participants’ perceptions of commercialization and abuse of FMT

After reading information about online patients’ postings about DIY FMT at home and Direct to Consumer (DTC) advertisement of FMT tool-kit and guidance book, more than half (56.1%) find this worrying because of unpredictable consequences. Of all respondents, 25.5% reported that DTC is common in other areas (e.g. genetic testing), and 18.4% reported no concerns.Following information about the possible role of gut microbiota on aging, obesity, immune system, when asked about application of microbiota-based therapy or synthetic microbiota in treating or enhancing some qualities (e.g.longevity) in the future, of all respondents, 77% predicted it is likely and may lead to safety-related risks, 17% reported unlikely, and 6% reported it is too far-fetched and imaginary.

### Participants’ beliefs about social and regulatory issues

74% participants agreed with the urgency to establish a standardized protocol of FMT which should be included in the governance and regulation of new technique by authorities, compared to 25.3% who believed the practice should be stopped, as the mechanism of FMT is still unknown. Comparison between clinicians who had high familiarity with FMT and those who had low familiarity (91.7% vs 64.1%, *p* = 0.005) is striking. A similar difference was found when asked if they agreed that FMT be recommended earlier as a first-line treatment option for CDI (rather than rescue last-resort therapy), 79.6% agreed vs. 20.4% who did not. An overwhelming majority (92.9%) indicated that fee standards of FMT should be established as soon as possible.

**Fig. 1:**
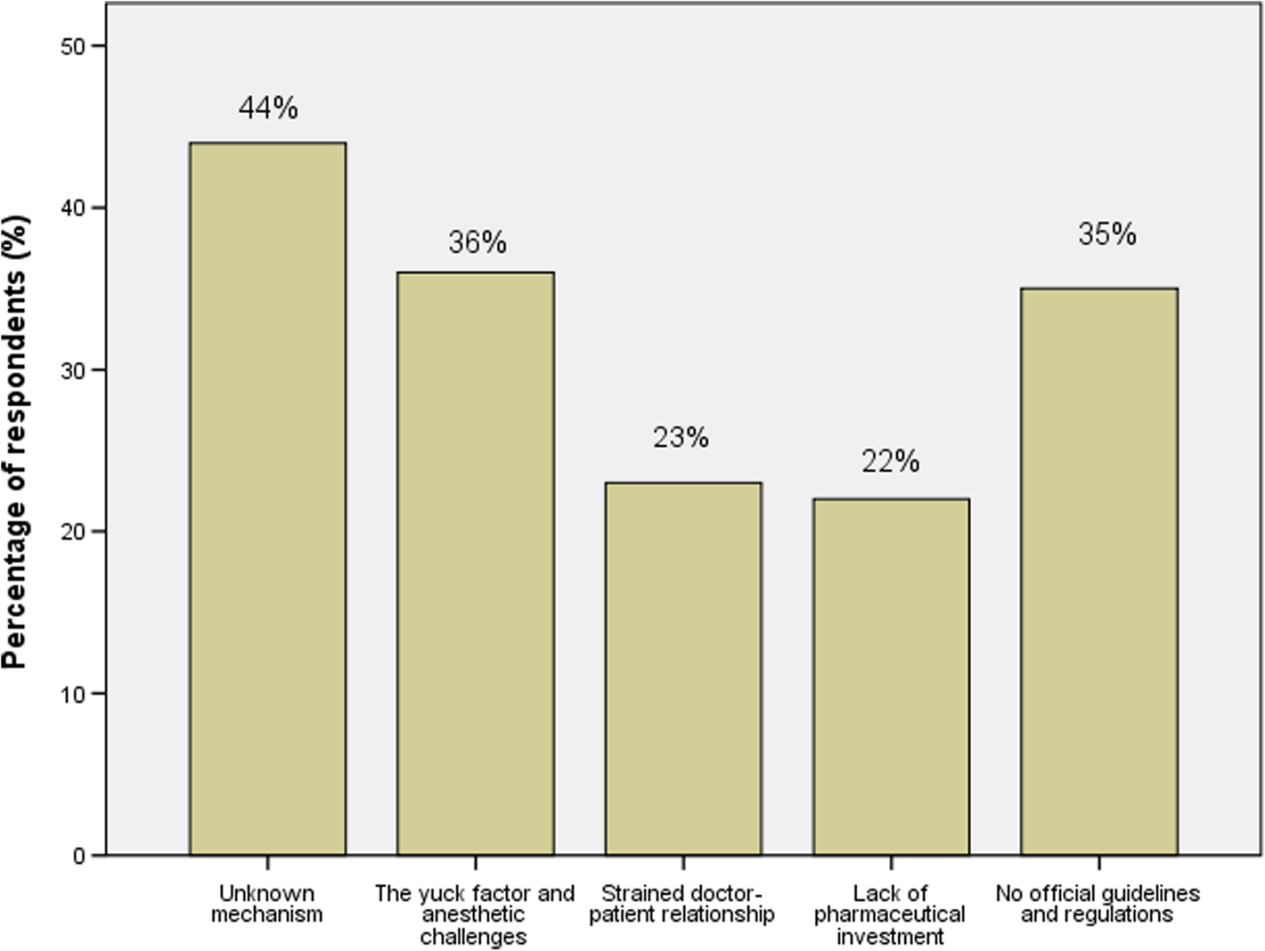
Participants’perceived barriers in promoting FMT

The proportion of clinicians reporting the greatest barriers to promotion of FMT are shown in Fig. 1.

### Participants’ views about fecal microbiota bank

**Fig. 2:**
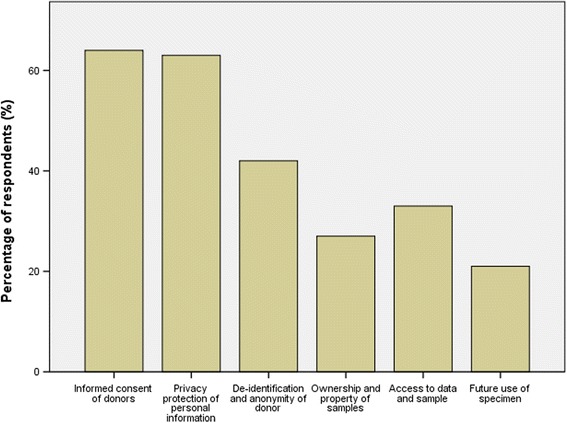
Participants’concerns about FMT bank

Regarding the ethical aspects of fecal microbiota banks, clinicians agreed informed consent of donors (64%), privacy protection of personal information (63%), de-identification and anonymity of donor (42%), ownership of samples (27%), access regulation to data and samples (33%), future use of samples (21%) were the most important factors in the construction of the banks (Fig. 2). Since a human microbiome bank may generate economic gain through marketing, when asked about justice issues in the allocation of burden and benefit, 63.6% respondents reported donors who contributed to research should receive benefit sharing, compared to 28.3% who indicated they should not receive this, and 8.1% who expressed no opinion.

## Discussion

The procedure of FMT occurs at the intersection of at least two ethically contentious areas: clinical research and therapy as well as donation and transplantation of renewable tissues (e.g. blood, bone marrow, semen) [14]. Each has a set of ethical and social implications which are already highly complicated, they are then further compounded by the practice of FMT. In relation to experimental treatment, FMT represents another therapeutic option to which patients/subjects who fail to respond to other treatments, may be driven by hope and desperation. This raises questions about the limitations of our knowledge and understanding of the new procedures, access to experimental treatment results as well as the extent of the risks involved. Especially the issues of selective release and/or publication of data related to clinical trials, with a bias towards positive results being available to healthcare professionals or the public. In relation to donation and transplantation of renewable tissues, unlike the self-limiting finite number of organs (e.g. kidney, liver), FMT is more like blood donation. An FMT donor can, after a suitable period to restore their biome, donate again. FMT shares some of the ethical concerns raised by the problem of supply (donor), demand (recipient), and appropriate allocation of scarce resources. Understanding the clinician’s perceptions and attitude is the key to improve healthcare quality and patients’ satisfaction.

### Low awareness but positive attitudes towards FMT

In this study, we found that the majority of clinicians (89%) are interested in and willing to consider performing FMT, despite the lack of adequate long-term safety and efficacy data. Interestingly, this study has also found that they have low familiarity and low awareness of the evidence in support of the use of FMT (64%). Our findings resonate with another recently published article, which reported that despite general poor health literacy on FMT, most surveyed Ontario physicians have shared similar positive attitudes about the effectiveness and safety of FMT [26]. It is worrisome, as clinicians’ enthusiasm for and willingness to provide FMT without a full understanding of the issue is potentially dangerous for patients, especially when long-term risk and safety are unknown. A possible explanation for Chinese clinicians’ positive attitudes is that both Traditional Chinese practitioners and Western-medicine trained doctors, have a general familiarity with the traditional folk practice of using “*jinzhi*” (golden soup), or “yellow dragon soup” (fecal suspension). In ancient China, when human fecal suspension was used as an effective treatment of diarrhea, vomiting, and constipation, the doctors labeled it as yellow soup (to avoid such responses and for aesthetic reasons), which were documented in “Ben Cao Gang Mu” (Compendium of Materia Medica) [7].

On the other hand, we also found clinicians who have negative attitudes towards some social media exaggerating and mystifying the effects of FMT as “magic” and “miraculous”, which may mislead patients. However, interestingly, nearly one third of the clinicians (30%) chose “more natural and organic” as the reason (next to effectiveness 88% and safety 64%) for recommending FMT to their patients, which may also be misleading in itself as “natural” and “organic” are never value-free words but rather commonly understood as the meaning of “safe” and “less risk” and somehow “healthier”. In medicine, “natural” is also often viewed as synonymous with “good” (and unnatural” as synonymous with “evil” or “bad”). This belief is problematic, for example, CDI is a natural phenomenon yet hardly considered good. If the clinician is unconsciously influenced by such emotive language, they may bias their advice to their patients towards acceptance of FMT without having a clear prior understanding of the medical, technical or ethical issues associated with the procedure. Subsequent failure of the intervention may skew perceptions of FMT’s actual effectiveness and unnecessarily prevent future patients from benefitting from FMT. Views about what is natural or unnatural may influence the degree to which technologies are embraced or opposed [34] by the public. Using this language and discourse surrounding FMT, patients may have unrealistically high expectation towards FMT while neglecting the risks. To illustrate, patients with Inflammatory Bowel Disease (IBD) may benefit from FMT and they are prime candidates for this treatment, but patients with acute or refractory IBD are particularly “vulnerable due to their healthcare experiences with ineffective therapies and subsequent poor quality of life, both of which may increase the propensity to make healthcare decisions based solely on desperation” [35]. An IBD patient’s autonomy may be compromised by their stress and desperation, affecting their ability to give informed consent. Undue influence and overhyped claims(from media and internet postings about DIY FMT) about the therapeutic effects of FMT may further complicate the IBD patient’s decision to seek treatment [14]. The clinicians fail to recognize the problems with their potentially misleading characterization/description of FMT as “natural” or “organic”, even as they criticize media representations of FMT as a miracle cure or otherwise exaggerate its effectiveness as a therapeutic intervention. This contradictory stance of clinicians also demonstrated their limited awareness of the literature of FMT, especially reports relating to risk and safety issues.

In one study, the validity and value of a clinical trial using FMT to treat ulcerative colitis was seriously compromised due to the high number of subject withdrawals (70%) [36]. The authors found that their participants experienced a therapeutic misconception due to the “failure to recognize the differences between clinical care and participation in a clinical trial, falsely believing that they will receive a direct clinical benefit”. The study demonstrated one potential pitfall of failing to consider the vulnerability of research participants. In particular, therapeutic misconception is more likely to occur in desperate or vulnerable populations [37], given the perception that FMT is a highly “sought-after innovative treatment”. The study would have benefited from greater attention to this potential problem from the outset. The lessons we learned from the ulcerative colitis study reinforces the need for informed consent to such a procedure where both clinician and patient are fully aware of the risks and benefits, and the clinician is honest in relation to their personal experiences with the procedure. Clinical audit is a valuable tool for the clinician in such discussions.

When it comes to the media coverage of FMT and its impact on patients’ decision making, it is useful if we compare it with other examples, e.g. gene therapy and stem cell treatment. Media using the words like ‘extremely promising’ and ‘cure’, as Caulfield and McGuire [38] pointed out, stem cell therapy is portrayed “in an uncritical manner and often as a cutting-edge therapy” and popular press tends to present unproven therapies “in a relatively positive light”. Moreover, for example, media portrayals of athletes and public figures will not only drive the market of stem cell therapies but also influence public opinion and science policy by implying the legitimacy of unproven and often unregulated treatments. Such rhetoric and media discourse contributes readily to therapeutic misconception.

At the same time, we found nearly half of the clinicians reporting “high expectation from patients and pressure on clinicians” (33%) as well as “not standard treatment, easily cause medical disputes” (14%) as their reasons for not referring for FMT, rather than the safety concerns. Our findings are similar to two other articles investigating clinicians’ attitudes toward FMT, one found “not having the right clinical situation” (33%) and “institutional or logistical barriers” (23%) as the main factors for not offering FMT [27]. The other paper found clinicians “unaware of where to access the treatment” and they “lacked knowledge on the treatment” as the top two reasons [26]. This demonstrated that contextual factors outside of the medical dimension of FMT need serious consideration.

### Clinician sensitivity to ethical concerns

Our study revealed that clinicians have different degrees of awareness and sensitivity to pre-identified ethical issues. This may depend on a combination and possible confliction between the clinician’s own moral code and beliefs with the requirements of professional ethics. We report three key ethical issues on which clinicians agreed on their importance: consent, privacy, and commercialization/abuse.

We found that most clinicians (80%) will provide patients with as much risk information related to FMT as possible, including the physical, mental, psychological changes, irrespective of the capacity of patients to understand. However, patients who desperately want FMT are exactly those who are more susceptible to harms from unknown/unsuspected side effects than others, whose vulnerability and compromised decision making capacity should be recognized and protected. Not surprisingly, the complexity of the disease and the motivation of the patient, may lead to conflict between a clinician’s therapeutic recommendations and the patient’s wishes. Some commentators proposed a so-called “treat-to-target” approach, that is, to set objective targets of disease control and serial adjustments to therapies, while enabling defined trials of alternative approaches, followed by a more objective assessment and reconsideration of treatments [39]. We believe this approach could be employed in consideration of FMT, respecting patient autonomy and the use of measurements of disease activity. Carefully shared decision-making about therapies, where the clinicians and patients share the best available evidence, and where patients are supported to consider options when faced with making decisions, will build trust in the physician-patient relationship. The over-riding concern for the clinician must always be to ‘do no harm’. In the case of FMT, there is insufficient information for the clinician to be sure the treatment work or that the patient will not get worse as a result of treatment. Hence, a cautious step-by-step approach using remedies whose success is well documented first would seem prudent, prior to employing remedies at the cutting edge of clinical research.

Doctors should convey to their patients that their uncertainty regarding risks and exploratory nature of the current FMT therapy option in a transparent, rational and non-directional manner, as this is a part of the informed consent process. Meanwhile, as Ma et al. suggested, some ethical guidance as to what would constitute appropriate information for informed consent to FMT would be helpful, in order for patients to make educated, autonomous decisions regarding their treatment [14].

With regards to stigma, we were surprised to find 43% of clinicians believed patients who undergo FMT may have “shame” or “degradation of dignity” versus 47% clinicians who did not think it is an issue as patients are terribly burdened by disease and these concerns were trivial compared to the suffering. Although these are disparate attitudes towards the “yuck factor” involved in FMT, they all suggest clinicians have certain level of empathy with the suffering of patients, either worry the patient’s acceptance of FMT, or have more deep understanding of the dilemma faced by patients, deciding between “unpleasant” FMT and enduring suffering caused by disease. This is further supported by the findings in this study that two thirds of the clinicians preferred colonic TET tube over nasogastric tube delivery, which is consistent with the results in many other surveys [15, 27, 28]. For example, in one study physicians predicted the most “negative scores” would be associated with receiving FMT through nasogastric tube while colonoscopy or enema would be “least unappealing” [27]. In the absence of information that shows whether the nasogastric method of delivery is more or less successful in managing the condition than the colonoscopy/enema route, it may be that the clinicians are basing their answers upon what would be their own preferences should they need this procedure. Their responses may be different of the FMT if it could be delivered by a less invasive route such as swallowable capsule rather than a nasogastric tube.

Schmidt [40] argued that the yuck factor reflects different layers of perception: at the emotional level, there are disgust and fear, while the more cognitive level is the repugnant feeling of moral violation. FMT can trigger responses in all three layers: disgust towards the object of feces, fear of transmission of potential pathogens in feces, and feelings of violation and degradation of human dignity by the use of FMT [14]. The findings by Kahn et al. suggest that branding and naming of FMT might be an important issue as some participants in their study expressed that the word fecal was off-putting. Moreover, several participants in their survey made comments that “they expected to hear jokes” if they told their family and friends about receiving fecal transplants [16]. As Chuong et al. pointed out, there are social stigma or concerns about facing stigma that could influence the treatment’s social acceptability [41]. Besides, cultural and religious beliefs about bodily integrity and dignity might also play a role which beyond the mere visceral “disgust and fear”, as some people may find receiving fecal transplant as “unsanitary” and “degraded”. For example, Muslim patients might have a strong prohibition against fecal transplant from non-Muslim donors. Or, vegan parents who might oppose FMT for their child from non-vegan donors because they might wish to raise their child in accordance with the same lifestyle; receiving fecal transplant defies that lifestyle and even dignity.

However, the “yuck factor” also opens up opportunities for dialogue between scientists, physicians, and the public regarding why a particular novel intervention should be pursued at all. Public reactions towards FMT often depend on where the information comes from and how it is presented. Physicians, policy makers, and the media have a duty to explain the pros and cons of FMT with supporting data in a transparent, responsible manner. Despite this, the volatility of public perception of what is ‘good’ or ‘bad’ can be heavily influenced by rogue individuals such as Andrew Wakefield, a gastroenterologist in the UK, who produced a paper in 1988 (later discredited) claiming a link between the MMR vaccination and subsequent development of autism and bowel disease [42]. His ideas were subsequently taken up in the social media by conspiracy theorists who invented their own ‘truths’.

A majority of clinicians were worried about the abuse of home DIY FMT (56.1%) and the commercialization of microbiome-based therapy, e.g. dietary supplements (77.1%). They particularly stressed the safety-related risks and unpredictable consequences. For example, one physician wrote short answers and mentioned a paper that reported the transfer of viral community between individuals [43]. However, 18.4% of all respondents reported no concerns, perhaps it is because these clinicians recognized the commercialization of FMT as a potential problem but not one that is likely to materialize. Clinicians welcome the establishment of a FMT bank as it not only shifts the burden of contact with stools, but also avoids the privacy problems.

Working out on how to define, identify and sourceoptimal donors has become a pressing clinical demand and a research area worthy of multidisciplinary investigation. It poses both medical and ethical challenges. Finding people who pass the donor screening criteria can be difficult as the strict and evolving screening standards present a severe constraint to donor numbers at present. An Australian study [44] showed that recruitment of fecal donors for an FMT bank is challenging, with only 2% of those enrolling, ultimately serving as donors. To relieve the burden associated with donor enrolment and stool preparation, stool banks have been established in some countries, for example, OpenBiome and AdvancingBio in the USA, the Taymount Clinic in the UK, the Netherlands Donor Feces Bank (NDFB), and the Chinese FMT bank.

Currently, a number of different FMT donor screening protocols have been published such as those of the FMT Working Group [45], the Joint Society Letter to FDA—Current Consensus Guidance on Donor Screening and Stool Testing for FMT [46], and the Amsterdam Protocol [47]. There are inconsistencies between the protocols in terms of requirements for particular tests as well as for the timing and interval frequency of such tests. Screening of potential donors and of stool and serum specimens is vital for preventing the transfer of infectious diseases and to mitigate the potential risksof making recipients more susceptible to chronic conditions such as obesity or autoimmune disorders [48]. As FMT banks currently remain limited to a few countries and medical institutions, those without access to such banks will lack a stable and continuous stool material and cannot schedule treatment in advance, so FMT is mostly performed on an ad-hoc basis.

### Limitations

This study had several limitations. Firstly, it is limited in size and scope: all subjects were from the Second China Enterology and FMT conference in 2015, therefore the findings may not be generalizable to other clinicians. Secondly, majority of clinicians were from tertiary teaching and academic medical institutions, so their knowledge and attitudes may not represent clinicians from community- and county-levels. However, the objective of this study was to assess clinicians’ perceptions of ethical and social concerns about FMT, and as such, sample size may have little relevance. Thirdly, this study was conducted involving clinicians of China, who have a specific cultural and economic background. Therefore, the respondents’ attitudes and perceptions may be reflective of that culture and may not be generalizable worldwide.

## Conclusion

This study provides the first assessment of clinicians’ perceptions and attitudes towards ethical and social challenges raised by FMT which must be addressed as part of a successful regulatory policy response. We found a majority of the surveyed clinicians have positive attitudes towards FMT but low awareness of knowledge and data. Our findings revealed the more common reasons for not offering or recommending FMT come from institutional, logistical and social factors, rather than the commonly perceived safety concerns. Patients’ consent and vulnerability, privacy and stigma, abuse and commercialization, were perceived as the most challenging ethical aspect of FMT. Clinicians are also greatly in favor of the establishment of fecal microbiota bank. This study indicates 1) the pressing need for regulating and standardizing FMT and determining its appropriate indications, 2) education and training for clinicians on FMT evidence base, and 3) risks which should be taken into account in FMT study design. More importantly, high level of trust should be built up in the physician-patient relationship prior to the procedures of FMT. Clinicians are responsible for cultivating this relationship of trust between themselves and their patients, this should include, e.g., demonstrations of trustworthiness on their part.

## Additional file

Additional file 1: Questionnaire: clinician's attitudes and perceptions of ethical and social issues in fecal microbiota transplantation. (DOCX 27 kb)

## Abbreviations

CDI: Clostridium Difficile infection
FDA: Food and Drug Administration
FMT: Fecal microbiota transplantation
IBD: Inflammatory bowel disease
