# Supplementary material for: How Chinese clinicians face ethical and social challenges in fecal microbiota transplantation: a questionnaire study
Source: BMC Med Ethics. 2017 May 31;18:39. doi: 10.1186/s12910-017-0200-2 (PMC5452366; doi:10.1186/s12910-017-0200-2)
Supplement: Additional file 1: — Questionnaire: clinician's attitudes and perceptions of ethical and social issues in fecal microbiota transplantation. (DOCX 27 kb) [file 12910_2017_200_MOESM1_ESM.docx]

**Questionnaire: attitudes and perceptions of ethical and social issues in**

**“Fecal microbiota transplantation (FMT)” No:______**

This survey aims to investigate your attitudes and perception of the ethical, legal, and social issues raised by Fecal microbiota tranpplantation (FMT). The study was initiated by Dr. Yonghui Ma from Medical College of Xiamen University, along with the Digestive Centre of Second Affiliated Hospital of Nanjing Medical University and the Department of Gastroenterology of Zhongshan Hospital affiliated of Xiamen University. This study has been approved by Institutional Review Board of these institutions respectively.

Your agreement to participate in this study is entirely voluntary. The information you provide will be used to help us improve our understanding of your concerns regarding FMT and to better inform policy makers to set up guidelines and regulations. Your agreement to participate in the study and completion of the questionnaire constitute your informed consent. Please also be assured that your data will be de-identified to protect your personal formation. Thank you for taking the time to complete the following questions.

If you have any questions, please contact Dr. Yonghui Ma: [yhma@xmu.edu.cn](mailto:yhma@xmu.edu.cn), or call: 0086-592-2185175

1. What is your profession?
2. Gastroenterologist and/or internists
3. nurse
4. microbiologists
5. pharmacologists
6. other profession

**General knowledge about FMT**

1. Before you participate in this conference, are you familiar with FMT?
2. Yes, I have also performed FMT
3. No, I am not familiar with FMT, but I have heard about it
4. No, I am not familiar nor heard about it
5. What do you think of FMT?
6. A promising treatment modality for some diseases
7. The efficacy and safety of FMT is very limited
8. The current data is not sufficient to support the use of FMT
9. If it is medical indicated and ethically approved, would you refer FMT to patient?
10. I will (please answer Q4)
11. I am not sure, it depends, e.g. conventional treatment fails. (please answer both Q4 and Q5)
12. I will not refer(please answer Q5)

**Ethical issues related to FMT**

Informed consent

1. What’s the reason for you to recommend FMT and which you will also inform patients?
2. Clinical efficacy
3. safety
4. failure of conventional treatment
5. more “natural” and “organic”
6. avoidance of antibiotics
7. What’s the reasons for you not to recommend FMT and which you will also inform patients?
8. Unproven treatment and unknown mechanism
9. infections
10. long-term risk and safety unknown
11. high expectation from patients puts pressure on physicians
12. not standard treatment, easily cause medical litigation
13. Some media describe FMT as “magic” and “miracle” therapy despite its unappealing nature, do you think this coverage have exaggerated the effect and downplayed the risk, and what impact on patient decision making?
14. I agree, this will mislead patients to unrealistic high expectations and neglect the risk
15. I do not agree, patients are capable of making autonomous decisions.
16. I do not know, patients would anyway be informed by the informed consent form

Risk, stigma and privacy issues

1. The risks of FMT includes not only physical adverse events, e.g. fever, abdominal cramping or constipation, elevation of C-reative protein unknown infections, but also the potential theoretical mental illness (transmission of anxiety and depression) and imbalance of neurotransmitters. Please choose the statement you agree.
2. I would inform patients all known and possible risks
3. I would only inform patients about the actual physical risk
4. The notification level of risk depends on the comprehensive capacity of patients
5. Do you believe accepting FMT has a negative impact on patient dignity (feelings of embarrassment, degradation, and stigma)?
6. Agree
7. Disagree, please indicate reasons______
8. I do not know
9. If upper (nasoduodenal/jejuna) and lower (transcolonoscopic/enema) are medically indicated, which do you think is the optimal modality to deliver FMT?

a) upper delivery

b) lower delivery

1. Regarding privacy issues in FMT, please select the following measures you think can protect privacy and confidentiality?
2. Both donor and recipient are double-blind
3. Donor should be anonymised
4. Establish standardized fecal microbiota bank
5. FMT patients should have private ward or room
6. Ensure confidentiality of patient information during communication with others

Commercialisation

1. There’s increasing online patients postings about DIY FMT at home and FMT tool-kit and guidance book, as well as Direct to Consumer (DTC) advertisement of fecal microbiota testing, what’s your attitude towards this phenomenon?
2. It is worrying because of unpredictable consequences.
3. DTC mode of healthcare service is common in other areas (e.g. genetic testing).
4. No concerns about this issue
5. As our understanding of the role of gut microbiota on aging, obesity, immune system advance, what do you think of the future application of microbiota-based therapy or synthetic microbiota in treating or enhancing some qualities (e.g. skin care)?
6. Very likely to happen but with safety and risk issues
7. Unlikely to happen
8. It is too far-fetched and imaginary

**Social and regulatory issues of FMT**

1. Do you believe there’s urgency to establish a standardized protocol of FMT and which should be included in the governance and regulation of new technique by authorities?
2. Yes, I agree
3. It should be suspended as the mechanism of FMT is still unknown
4. Do you agree that charging standard of FMT should be established?
5. Yes, it should be fixed ASAP.
6. No need at the moment.
7. Do you agree that FMT should be recommended earlier as first-line treatment option (rather than rescue last-resort therapy) for CDI?
8. Yes, I agree
9. No, I donot agree
10. Please choose the factor(s) you believe can hinder the promotion of FMT?
11. Unknown mechanism
12. The yuck factor and anesthetic challenges
13. Stained doctor-patient relationship
14. Lack of pharmaceutical investment
15. No official guidelines and regulations

**Fecal microbiota bank**

1. What ethical aspect(s) concerns you most, if any, regarding fecal microbiota bank? (you could choose 3 options maximum)
2. Informed consent mode
3. Privacy protection of personal information
4. De-identification and anonymity of donors
5. Ownership and property of samples
6. Access regulation to data and sample
7. Future use of specimen and re-contact
8. Since human microbiota bank may generate economic gains through patenting and marketing, what do you think of justice issues in the allocation of burden and benefit?
9. Donors who contributed to research should receive benefit sharing
10. Donors should not receive benefit sharing
11. I do not care about this issue.
12. Any other comments regarding FMT that you wish to make?

____________________________________________________________________________________________________________________________________________________________________________________________________________________________________________________________________________________________________________
